# Supplementary figures and images for: Examining the associations between a generalist feeder and a highly toxic host
Source: Ecol Evol. 2024 Feb 21;14(2):e11035. doi: 10.1002/ece3.11035 (PMC10880132; doi:10.1002/ece3.11035)

**Negative Control (Tomato) Media**

**Female Fly**

**Edible Mushroom Media**

**Toxic Mushroom Media**

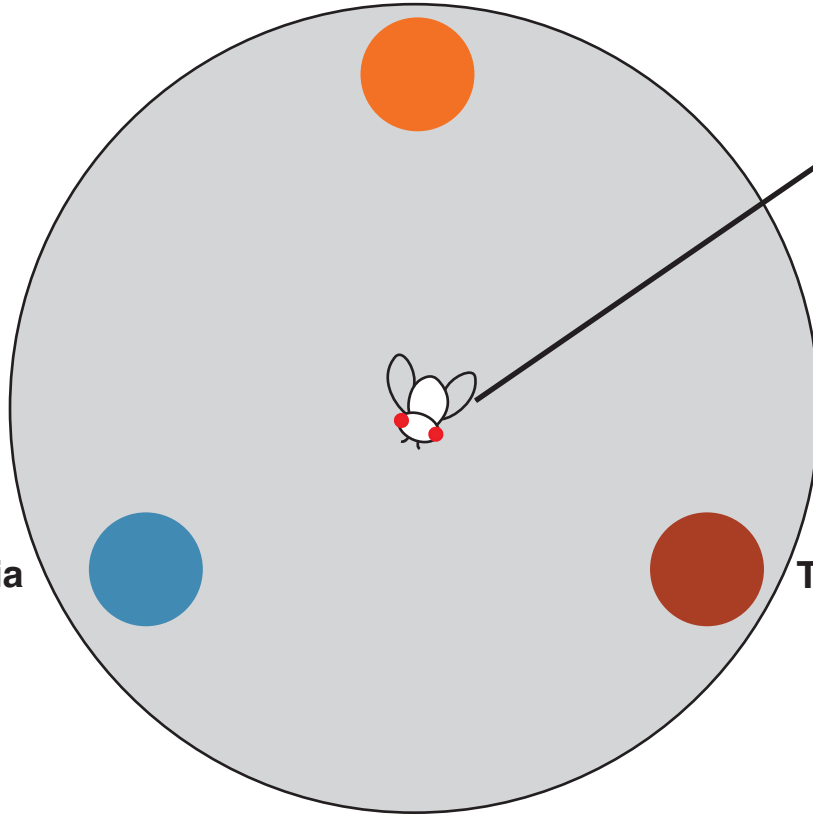

Supplement: Supplementary file 2 — Figure S1. [file ECE3-14-e11035-s003.pdf]

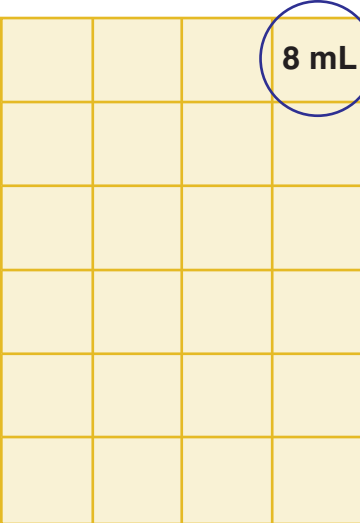

8 mL

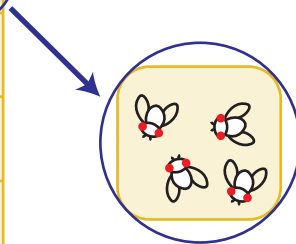

14 Hours

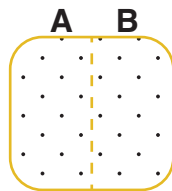

Media Cut Here

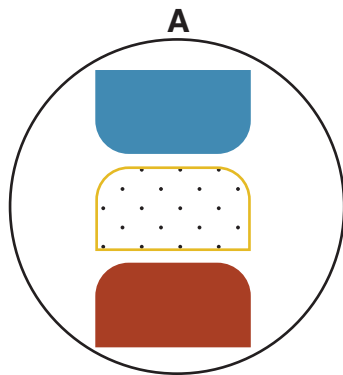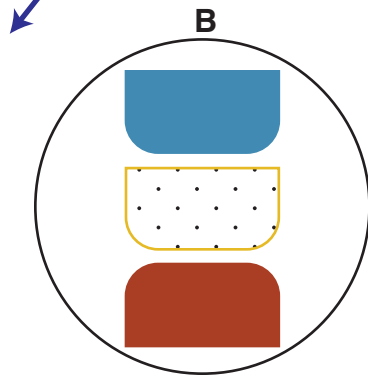

Supplement: Supplementary file 3 — Figure S2. [file ECE3-14-e11035-s002.pdf]
